# Tornado Intensity Estimated from Damage Path Dimensions

**DOI:** 10.1371/journal.pone.0107571

**Published:** 2014-09-17

**Authors:** James B. Elsner, Thomas H. Jagger, Ian J. Elsner

**Affiliations:** 1 Department of Geography, Florida State University, Tallahassee, FL, United States of America; 2 Digital Worlds Institute, University of Florida, Gainesville, FL, United States of America; Fondazione Edmund Mach, Research and Innovation Centre, Italy

## Abstract

The Newcastle/Moore and El Reno tornadoes of May 2013 are recent reminders of the destructive power of tornadoes. A direct estimate of a tornado's power is difficult and dangerous to get. An indirect estimate on a categorical scale is available from a post-storm survery of the damage. Wind speed bounds are attached to the scale, but the scale is not adequate for analyzing trends in tornado intensity separate from trends in tornado frequency. Here tornado intensity on a continuum is estimated from damage path length and width, which are measured on continuous scales and correlated to the EF rating. The wind speeds on the EF scale are treated as interval censored data and regressed onto the path dimensions and fatalities. The regression model indicates a 25% increase in expected intensity over a threshold intensity of 29 m s^−1^ for a 100 km increase in path length and a 17% increase in expected intensity for a one km increase in path width. The model shows a 43% increase in the expected intensity when fatalities are observed controlling for path dimensions. The estimated wind speeds correlate at a level of .77 (.34, .93) [95% confidence interval] with a small sample of wind speeds estimated independently from a doppler radar calibration. The estimated wind speeds allow analyses to be done on the tornado database that are not possible with the categorical scale. The modeled intensities can be used in climatology and in environmental and engineering applications. Research is needed to understand the upward trends in path length and width.

## Introduction

A tornado is a violently rotating column of air capable of producing catastrophic damage where it comes in contact with the ground. The United States experiences more tornadoes than any country on earth [1]. Advances in technology have improved forecasts and warnings of these events; nevertheless, the active 2011 season (with over 1700 tornadoes) took the lives of more than 550 people [2]. The devastating impacts from these events [3] make understanding and predicting them important. But the short duration and unpredictable nature of tornadoes together with extreme velocities make it difficult to obtain direct measurements of wind speeds within the vortex.

Post storm surveys of the destruction in the wake of a tornado allow engineers to rate the damage on a scale from zero to five. Historically the damage scale was related physically to the tornado wind speed [4], [5]. Today wind speed is phenomenologically related to the observed damage [6]. The estimated wind speed is a 3 sec gust at the location of damage based on indicators of damage to structures and vegetation and includes the degree of damage taking into account differences in construction quality [7]. For instance EF1 (category one on the Enhanced Fujita scale) damage corresponds to wind speeds between 38 and 49 m s^−1^ and EF4 damage corresponds to wind speeds between 75 and 89 m s^−1^ (derived EF scale). The EF rating assigned to tornadoes in the historical record is the highest damage category found within the damage path [8]. The EF scale is consistent with the original F scale but it includes additional damage indictors and it expands on the degree of damage. The scale was formally adopted by the U.S. National Weather Service (NWS) in 2007. Studies have addressed the need for more reliable measures of tornado winds and the potential discrepancies between wind speeds estimated by radar and damage ratings [9].

The damage rating is correlated to path length and path width [10]. Here we make use of these relationships to build a statistical model for a single ‘best’ estimate of tornado intensity. We do this by assuming a Weibull distribution for intensity and by treating the wind speed ranges on the EF scale as interval-censored data. The latter means the unobserved intensity of the tornado falls somewhere between the end points of the interval defined by each EF category. The model provides an estimate of the intensity (highest) on a continuous scale for each tornado in the database allowing climatologists to examine changes in intensity separate from changes in frequency. This is crucial for understanding the relationship between tornadoes and climate change because increasing intensity does not need to imply more strong tornadoes if frequency decreases. Moreover, intensities together with some additional assumptions can be used to estimate tornado power. This is important as results from climate models indicate that the future might include more days when high wind shear coincides with high values of convective available potential energy [11] suggesting perhaps the possibility of more powerful tornadoes.

The paper is outlined as follows. In section 2 we briefly describe the data used in this study and examine damage path relationships. Our focus is on the most recent set of years when the quality and consistency of reporting is at its highest. In section 3 we describe our statistical model for tornado intensity. We examine model fit and provide an interpretation of the coefficients. In section 4 we examine model validity and adequacy. We also consider the variation in annually-averaged tornado intensity. In section 5 we give a brief summary and provide some concluding remarks. The code to reproduce the analysis and results is available at rpubs.com/jelsner/TornadoIntensityModel.

## Methods

### Tornado Damage Path Relationships

The U.S. Storm Prediction Center (SPC) maintains the most up-to-date and readily available record of tornadoes in the United States compiled from NWS *Storm Data* publications and reviewed by the U.S. National Climate Data Center [12]. We obtain the dataset containing all reported tornadoes over the period 1950–2013 from www.spc.noaa.gov/gis/svrgis/ and subset for years beginning with 2007 when the EF scale was was adopted. According to a report by the Pacific Northwest National Laboratory for the U.S. Nuclear Regulatory Commission, the SPC database is in reasonably good condition and acceptable for use in this type of analysis [13].

We consider all tornadoes with an EF rating for a total of 8,752 over the period 2007–2013, inclusive. Damage assessments are made using radar tracking, eyewitness accounts, media reports, and damage photos and videos. Sometimes areal and ground surveys are taken. If a tornado produces at least one fatality, numerous injuries requiring hospitalization, extensive property damage, or widespread media interest (defined as a significant event), the damage rating is determined by meteorologists and engineers after a ground and/or aerial survey. Besides the EF rating the damage assessment includes the path length and maximum path width. There were 4994 EF0, 2642 EF1, 818 EF2, 232 EF3, 57 EF4 and 9 EF5 tornado reports during the period of study and there are no significant upward or downward trends in the annual frequencies by damage rating. The average number of tornadoes per year over this period is 1250.

**Figure 1:**
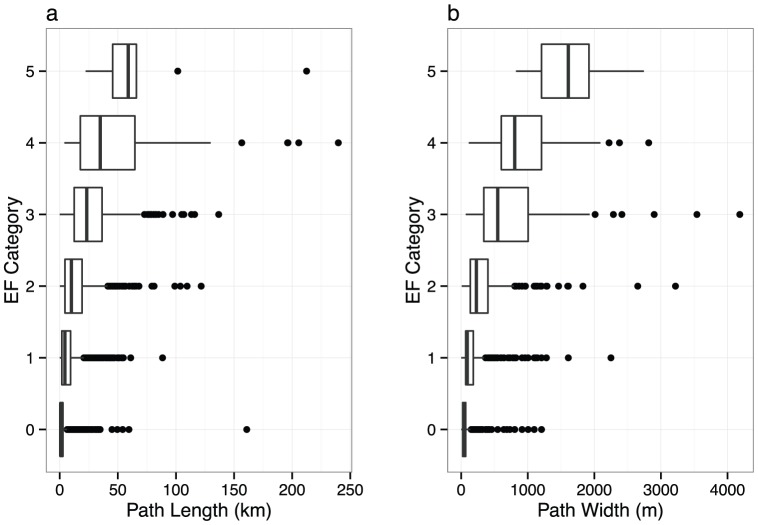
Box plots of damage path length (a) and path width (b) by EF category.

Damage path length and width are related to EF rating [10] as shown in Table 1. Lengths are recorded to the nearest hundredth of a mile and widths to the nearest yard but frequently rounded to the nearest 5 or 10 yards. The width represents the widest extent of the path. We assume that the path does not include damage associated with the rear-flank downdraft. The number of violent tornadoes (EF4 and EF5) is only .7% of the total number of all tornadoes. The total range of path length and width is large even within individual EF categories. However there is a clear relationship between EF category and path length as well as between EF category and path width (Fig. 1).

**Table 1:** Damage path statistics.

Category	Wind Speed	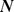	Length (km)		Width (m)	
	(m s^−1^)		Mean	Median	Mean	Median
EF0	[29–38]	4994	2.27	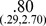	54.9	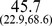
EF1	[38–49]	2642	7.10	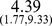	163.8	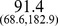
EF2	[49–62]	818	14.29	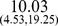	344.1	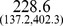
EF3	[62–75]	232	29.09	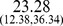	736.3	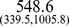
EF4	[75–89]	57	52.55	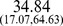	997.9	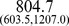
EF5	[89–105]	9	71.95	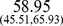	1635.8	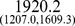

The derived EF scale and corresponding wind speed ranges based on three second gusts. Data are based on all reported tornadoes in the United States (2007–2013). 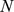 is the sample size. The lower and upper quartile values are given in parentheses.

Brooks [10] fits Weibull distributions to path length and path width by damage rating. Here we show the distributions of actual length and width since we use them as covariates in a statistical model describing their relationship with EF rating. We note that mean path length and width have increased over time so the values in Table 1 are larger than the corresponding values reported earlier in [10] based on data over the period 1950–2001. Length explains 30% of the variability in EF rating and width explains 37% of the variability. Width explains 35% of the variability in length.

### Weibull regression

Here we exploit these relationships in a statistical model for tornado intensity by assuming a Weibull distribution for the intensity and by treating the wind speed ranges on the derived EF scale as censored interval data (see Table 1). The Weibull distribution has previously been used to model the highest wind speeds associated with hurricanes [14]. The model assumes independent observations, which is reasonable given the discrete nature of tornadoes in space and time. The location and scale parameters are modeled separately. The model for the location parameter 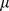 has the form



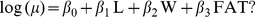
(1)where L is path length, W is path width, and FAT? is whether or not there was at least one fatality. A maximized likelihood procedure is used to fit the model [15] and to obtain the coefficients (Table 2). The model that includes the fatality term has lower AIC (14972) than the one without it (15024).

**Table 2:** Table of model coefficients.

log( 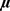 ) Parameters				
Covariate	Estimate	Standard Error (S.E.)	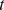 value	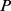 -value
(Intercept)	2.052	7.413 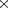 10^−3^	276.809	0
Length	2.265 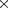 10^−5^	9.347 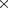 10^−7^	24.238	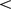 .0001
Width	1.544 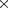 10^−3^	4.419 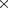 10^−5^	34.944	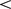 .0001
FAT? (yes)	.3586	.0551	6.508	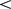 .0001
log( 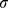 ) Parameters				
(Intercept)	.6324	9.297 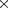 10^−3^	66.022	0
Length	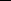 3.117 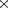 10^−4^	4.033 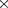 10^−5^	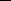 7.730	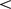 .0001
Width	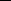 4.044 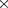 10^−6^	8.474 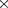 10^−7^	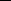 4.772	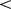 .0001

The model has a location [log(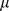)] and a scale [log(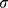)] component.

## Results

The model quantifies the significant relationship between damage-rating wind speed intervals and path length and width. Model coefficients are ratios based on the exceedance over the threshold of 29 m s^−1^ (lower bound on the EF0 rating). The coefficient on the length term is 2.265
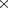
10^−5^ so 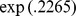 = 1.25 or a 25% increase over the threshold for a 100 km increase in path length. The coefficient on the width term is 1.544
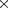
10^−3^ so 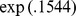 = 1.17 or a 17% increase over the threshold for a 1 km increase in path width. The fatality term is significant and indicates a 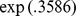 = 1.43 or a 43% increase in the expected intensity when fatalities are observed. That is, on average, tornadoes that kill have been about 43% stronger than those that did not.

For a fixed variance, a 50 m s^−1^ ten km long tornado will on average be a (50
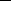
29)
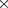
1.25
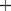
29 = 55.3 m s^−1^ tornado and a 50 m s^−1^ 100 m wide tornado will be a (50–29)
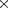
1.17
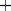
29 = 53.6 m s^−1^ tornado. A 50 m s^−1^ tornado observed with no fatalities with the same width and length would be a (50–29)
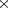
1.43
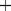
29 = 59.0 m s^−1^ tornado. The Weibull shape parameter (sigma) is allowed to vary with length and width in the model, but the coefficients on this term are about an order of magnitude smaller than the coefficients on the mean term. For instance, 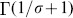 = .891 for a 100 m wide, 10 km long tornado and .895 for a 200 m wide, 20 km long tornado. This is a change of .4%, so we can ignore this variation when interpreting the results, although we note that the signs on the length and width coefficients in the model for the scale parameter are negative indicating that the shape parameter decreases for larger widths and lengths. The shape of the intensity distribution becomes broader as tornado path width and length increase.

**Figure 2:**
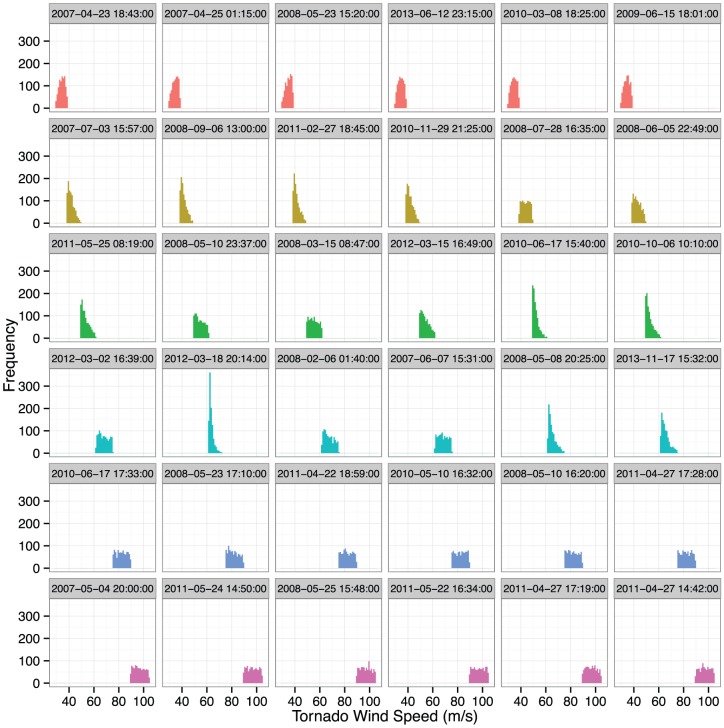
Histograms of predicted tornado intensities for 36 tornadoes since 2007. Six randomly chosen from each of six EF ratings (top row lowest to highest). Date/time are given in the panel heading.

Given an EF rating along with path length, path width, and whether or not there was a fatality, the model generates samples of predictive intensity (Fig. 2). The plot shows histograms based on 1000 samples from 36 tornadoes since 2007. Six randomly chosen tornadoes are included from each of the six EF ratings (top row lowest to highest) with the date/time displayed in the panel heading. The histograms are bounded by the wind speeds assigned to the EF category. In some cases the histogram is flat indicating that the length and width do not provide information on tornado intensity beyond the EF rating. However there are exceptions especially for tornadoes with ratings between EF1 and EF3. Here we see cases where the distribution is positively skewed indicating that length and width suggest a lower-end intensity for the given rating.

### Adequacy and validation

**Figure 3:**
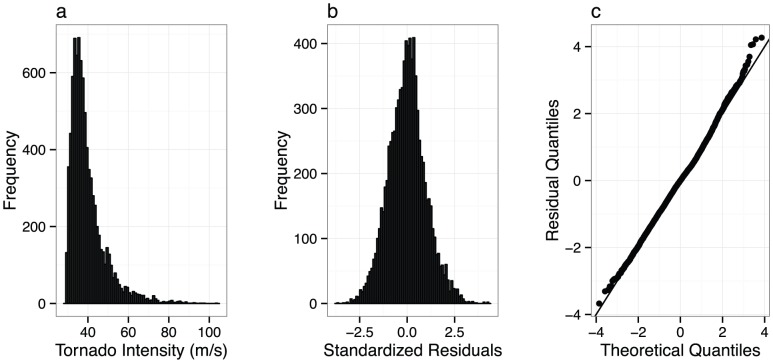
Model diagnostic plots. (a) Histogram of predicted tornado intensities. (b) Histogram of model residuals. (c) Quantile-normal plot of the model residuals.

The collective predictive distribution and model residuals are used to check against model adequacy (Fig. 3). The shape of the predictive distribution appears reasonable for intensity with a positive skew and a long right tail. There is a small notch in the distribution around the cutoff between EF1 and EF2 tornadoes. This is explained by relatively few high-end EF1s and relatively many low-end EF2s predicted based on path dimensions. The model residuals are approximately normally distributed with the exception being a long right tail indicating a few unusually short-lived and narrow tornadoes relative to their high assessed EF rating.

**Figure 4:**
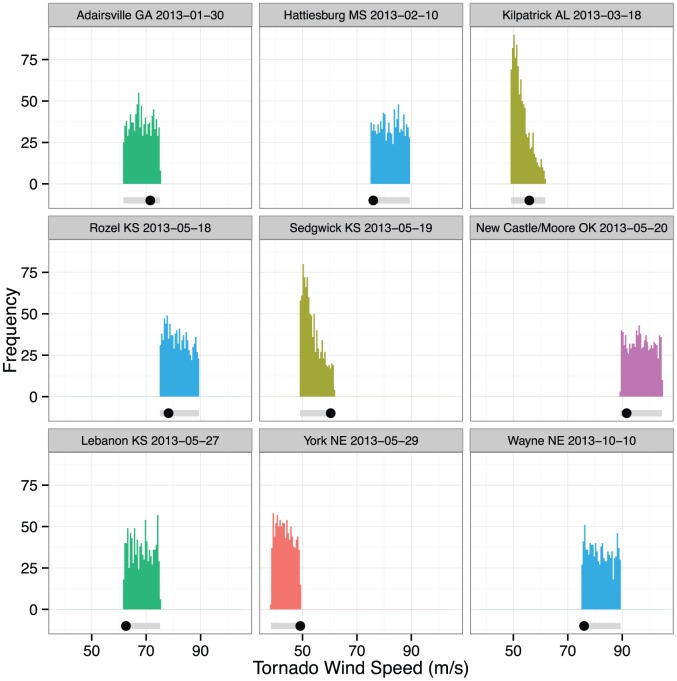
Histograms of predicted tornado intensities for nine tornadoes from 2013 for which a wind speed was estimated. The location of the estimated wind speed is shown as a dot and the range of wind speeds defined by the corresponding EF category is shown as a gray horizontal bar.

Individual predictive distributions provide a check on model validity. The vast majority of tornadoes do not have an estimated wind speed. However, in some cases it is possible to obtain a wind speed estimate from a nearby Weather Service Radar-88D measurements or from a mobile radar (Doppler on Wheels). We obtain estimates from the SPC's *Storm Reports* for nine tornadoes during the 2013 season and compare them to samples from our intensity model (Fig. 4). The wind speeds range from a low of 56 m s^−1^ for the Kilpatrick, AL March 18th EF2 tornado to a high of 92 m s^−1^ for the New Castle/Moore OK May 20th EF5 tornado. The predictive distributions are shown as histograms. In cases where the histograms are skewed, the estimated wind speed tends to be on the corresponding side of the EF range. The exception is the Sedgwick, KS May 19th tornado. The correlation between the estimated wind speeds and the average over all predictive samples is .98 with a root-mean squared error of 5.4 m s^−1^.

Model skill is estimated relative to a null model of choosing a random intensity within the wind speed ranges. Skill is assessed as the percentage increase in the coefficient of variation between path dimension and predicted intensities. The correlation between path length and EF rating is .552. The correlation between length and tornado intensity using our intensity model is .604 for an increase in the coefficient of variation of 20%. The correlation between length and tornado intensity from the null model is .566 for an increase in the coefficient of variation of 5%. Similar skill metrics are noted using path width.

We perform an out-of-sample test by correlating the modeled intensities from twelve tornadoes that have corresponding wind speeds estimated from radar measurements that are independent of the damage assessment. The derived radar wind speeds result from a calibration of mobile radar (Doppler on Wheels) with nearby Weather Service Radar-88D measurements. The data and method used to obtain the radar wind speeds are described in [16]. The modeled intensities correlate with the radar wind speeds at .77 (.34, .93) [95% CI]. Although this is a small sample of tornadoes the radar-estimated wind values range from a low of 38 m s^−1^ to a high of 91 m s^−1^ suggesting the potential for our estimates to be broadly applicable throughout the database.

**Figure 5:**
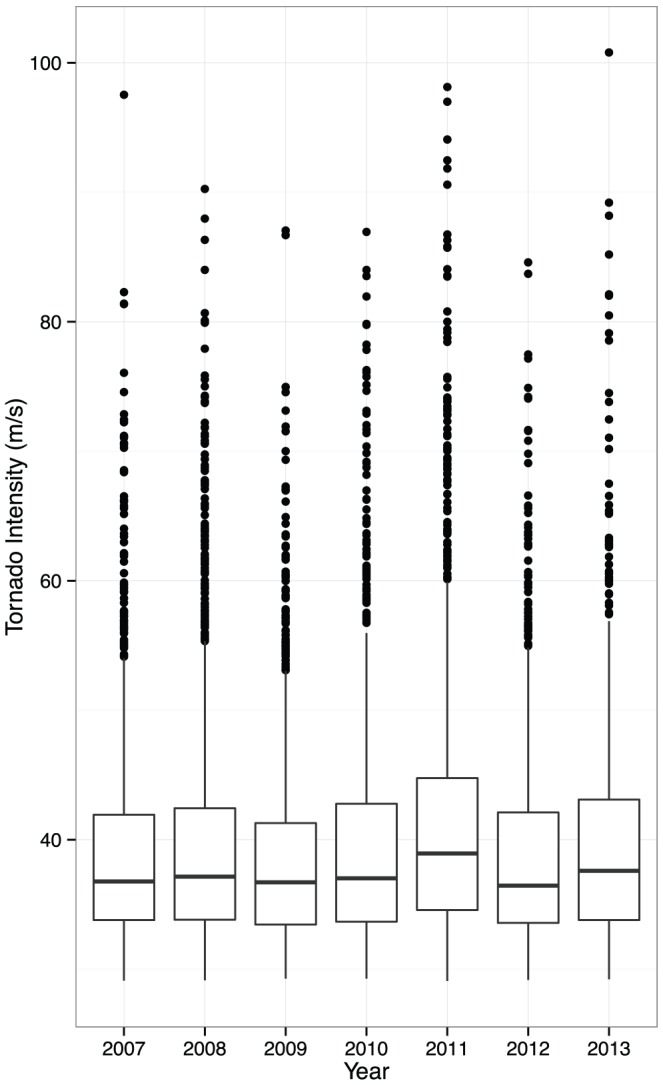
Box plots of predicted tornado intensity by year.

Finally a single predictive sample for each tornado is plotted by year as a box plot (Fig. 5). While the number of years is too few to ascertain a significant trend, there is an apparent increase in the upper quantiles of the annual intensity distributions resulting from the noted increases in damage path dimensions. Tornado intensity depends on updraft speeds within the parent thunderstorm and on increasing winds with height (shear) in the environment surrounding the thunderstorm [17]. Updraft speed is directly related to the available potential energy in the environment, which increases with greater low altitude heat and moisture. Upward trends in surface dew point temperature and specific humidity across the United States are coincident with upward trends in temperature especially over the tornado-prone Midwest [18]. With all else equal greater surface humidity implies greater available potential energy. Local shear can be large, even if it decreases in the mean, when waves in the upper-level flow amplify as occurs more often when warming in the Arctic outpaces warming elsewhere [19].

## Discussion

Tornadoes are capable of catastrophic damage. The Newcastle/Moore, OK tornado of May 20, 2013 and the El Reno, OK tornado just a week later are some recent examples. Direct measurements of tornado intensity are difficult and dangerous to get. Surveys rate the tornado damage on the EF scale and wind speed bounds are attached to the scale. Unfortunately the categorical scale is not adequate for analyzing tornado intensity separate from tornado frequency. Moreover, the historical database cannot directly benefit from improved surveillance technology.

Here we use path length and width which are measured on a continuous scale and which are strongly correlated to the EF category to estimate tornado intensity on a continuum. The model indicates a 25% increase in expected intensity over a threshold intensity of 29 m s^−1^ for a 100 km increase in path length and a 17% increase in expected intensity over the threshold for a 1 km increase in path width. The model also indicates a 43% increase in the expected intensity when fatalities are observed holding path dimensions constant. Diagnostic plots of the predictive density and residuals reveal no significant concern about model adequacy.

The modeled intensity allows analyses to be done on the tornado database not possible with the categorical scale. The predicted probabilities by EF category can be calibrated to the area affected by this level of damage and an index of tornado destructiveness would then follow naturally. The modeled intensities can be used in climatology and in environmental and engineering applications but more work needs to be done to understand the reason behind the increase in path length and width.

## References

[1] Grazulis TP (1990) Significant Tornadoes, 1880–1989: Discussion and analysis. Significant Tornadoes, 1880–1989. Environmental Films. URL http://books.google.com/books?id=E8hFAAAAYAAJ.

[2] Simmons KM, Sutter D (2012) The 2011 tornadoes and the future of tornado research. Bulletin of the American Meteorological Society 93: 959–961.

[3] Brooks H, Doswell C (2001) Normalized damage from major tornadoes in the United States: 18901999. Weather and Forecasting 16: 168–176.

[4] Fujita T, Pearson AD (1973) Results of FPP classification of 1971 and 1972 tornadoes. In: Eight Conference on Severe Local Storms. pp. 142–145.

[5] Fujita TT (1981) Tornadoes and downbursts in the context of generalized planetary scales. Journal of Atmospheric Science 38: 1511–1534.

[6] Feuerstein B, Dotzek N, Grieser J (2005) Assessing a tornado climatology from global tornado intensity distributions. Journal of Climate 18: 585–596.

[7] Edwards R, LaDue JG, Ferree JT, Scharfenberg K, Maier C, et al (2013) Tornado intensity estimation: Past, present, and future. Bulletin of the American Meteorological Society 94: 641–653.

[8] Doswell CA, Brooks HE, Dotzek N (2009) On the implementation of the enhanced Fujita scale in the USA. Atmospheric Research 93: 554–563.

[9] Wurman J, Alexander CR (2005) The 30 May 1998 Spencer, South Dakota, storm. Part II: Comparison of observed damage and radar-derived winds in the tornadoes. Monthly Weather Review 133: 97–119.

[10] Brooks HE (2004) On the relationship of tornado path length and width to intensity. Weather and Forecasting 19: 310–319.

[11] Diffenbaugh NS, Scherer M, Trapp RJ (2013) Robust increases in severe thunderstorm environments in response to greenhouse forcing. Proceedings of the National Academy of Sciences. 10.1073/pnas.1307758110 PMC3799355 24062439

[12] Doswell CA, Burgess DW (1988) On some issues of United States tornado climatology. Monthly Weather Review 116: 495–501.

[13] Ramsdell JV Jr, Rishel JP (2007) Tornado Climatology of the Contiguous United States. Technical Report NUREG/CR-4461, PNNL-15112, Pacific Northwest National Laboratory, P.O. Box 999, Richland, WA 99352.

[14] Jagger TH, Elsner JB, Niu XF (2001) A dynamic probability model of hurricane winds in coastal counties of the United States. Journal of Applied Meteorology 40: 853–863.

[15] Rigby RA, Stasinopoulos DM (2005) Generalized additive models for location, scale and shape,(with discussion). Applied Statistics 54: 507–554.

[16] Toth M, Trapp RJ, Wurman J, Kosiba KA (2013) Comparison of mobile-radar measurements of tornado intensity with corresponding WSR-88D measurements. Weather and Forecasting 28: 418–426.

[17] Trapp RJ, Diffenbaugh NS, Brooks HE, Baldwin ME, Robinson ED, et al (2007) Changes in severe thunderstorm environment frequency during the 21st century caused by anthropogenically enhanced global radiative forcing. Proceedings of the National Academy of Sciences 104: 19719–19723.

[18] Brown PJ, DeGaetano AT (2013) Trends in U.S. surface humidity, 1930–2010. Journal of Applied Meteorology and Climatology 52: 147–163.

[19] Francis JA, Vavrus SJ (2012) Evidence linking arctic amplification to extreme weather in midlatitudes. Geophysical Research Letters: L06801.

